# Machine Learning Assisted Design of Highly Active Peptides for Drug Discovery

**DOI:** 10.1371/journal.pcbi.1004074

**Published:** 2015-04-07

**Authors:** Sébastien Giguère, François Laviolette, Mario Marchand, Denise Tremblay, Sylvain Moineau, Xinxia Liang, Éric Biron, Jacques Corbeil

**Affiliations:** 1 Department of Computer Science and Software Engineering, Université Laval, Québec, Canada; 2 Department of Biochemistry, Microbiology and Bioinformatics, Université Laval, Québec, Canada; 3 Faculty of Pharmacy, Université Laval, Québec, Canada; 4 Department of Molecular Medicine, Université Laval, Québec, Canada; University of Toronto, CANADA

## Abstract

The discovery of peptides possessing high biological activity is very challenging due to the enormous diversity for which only a minority have the desired properties. To lower cost and reduce the time to obtain promising peptides, machine learning approaches can greatly assist in the process and even partly replace expensive laboratory experiments by learning a predictor with existing data or with a smaller amount of data generation. Unfortunately, once the model is learned, selecting peptides having the greatest predicted bioactivity often requires a prohibitive amount of computational time. For this combinatorial problem, heuristics and stochastic optimization methods are not guaranteed to find adequate solutions. We focused on recent advances in kernel methods and machine learning to learn a predictive model with proven success. For this type of model, we propose an efficient algorithm based on graph theory, that is guaranteed to find the peptides for which the model predicts maximal bioactivity. We also present a second algorithm capable of sorting the peptides of maximal bioactivity. Extensive analyses demonstrate how these algorithms can be part of an iterative combinatorial chemistry procedure to speed up the discovery and the validation of peptide leads. Moreover, the proposed approach does not require the use of known ligands for the target protein since it can leverage recent multi-target machine learning predictors where ligands for similar targets can serve as initial training data. Finally, we validated the proposed approach in vitro with the discovery of new cationic antimicrobial peptides. Source code freely available at http://graal.ift.ulaval.ca/peptide-design/.

## Author Summary

Part of the complexity of drug discovery is the sheer chemical diversity to explore combined to all requirements a compound must meet to become a commercial drug. Hence, it makes sense to automate this chemical exploration endeavor in a wise, informed, and efficient fashion. Here, we focused on peptides as they have properties that make them excellent drug starting points. Machine learning techniques may replace expensive *in-vitro* laboratory experiments by learning an accurate model of it. However, computational models also suffer from the combinatorial explosion due to the enormous chemical diversity. Indeed, applying the model to every peptides would take an astronomical amount of computer time. Therefore, given a model, is it possible to determine, using reasonable computational time, the peptide that has the best properties and chance for success? This exact question is what motivated our work. We focused on recent advances in kernel methods and machine learning to learn a model that already had excellent results. We demonstrate that this class of model has mathematical properties that makes it possible to rapidly identify and sort the best peptides. Finally, *in-vitro* and *in-silico* results are provided to support and validate this theoretical discovery.

## Introduction

Drug discovery faces important challenges in terms of cost, complexity and the amount of time required to yield promising compounds. To avoid side effects, a valuable drug precursor must have high affinity with the target protein while minimizing interactions with other proteins. Unfortunately, only a few have such properties and these have to be identified from an astronomical number of candidate compounds. Other factors, such as bioavailability and stability have to be considered; but this combinatorial search problem, by itself, is very challenging [1].

For novel and less studied targets, screening compound libraries remain the method of choice for rapid data generation. To fully exploit the great conformational and functional diversity, combinatorial peptide chemistry is certainly a powerful tool [2–4]. A major advantage of using combinatorial peptide libraries over classic combinatorial libraries, where the scaffold is fixed, is the possibility of generating enormous conformational and functional diversity using a randomized synthesis procedure. This chemical diversity and functionality can be further enhanced by the inclusion of non-natural amino acids [5]. Furthermore, having a peptide scaffold can be very informative to screen for similarities in peptidomimetic libraries [6]. For these reasons, this work will focus on using peptides as drug precursors.

However, it is important to note that combinatorial peptide chemistry cannot cover a significant part of the peptide diversity when peptides are longer than a few amino acids. For example, 2*g* of a one-bead one-compound (OBOC) combinatorial library [7] composed of randomly-generated peptides of nine residues will generate a maximum of six million compounds, representing a vanishingly small fraction (less than 0.0016%) of the set of all 20^9^ peptides. Consequently, it is almost certain that the best peptides will not be present and most synthesized peptides will have low bioactivity. Hence, drug discovery is a combinatorial problem which, unfortunately, cannot be solved using combinatorial chemistry alone. The process of discovering novel compounds with both high bioactivity and low toxicity must therefore be optimized.

Machine learning and kernel methods [8] have the potential to help with this endeavour. These algorithms are extremely effective at providing accurate models for a wide range of biological and chemical problems: anti-cancer activity of small molecules [9], protein-ligand interactions [10] and protein-protein interactions [11]. The inclusion of similarity functions, known as *kernels* [8], provides a novel way to find patterns in biological and chemical data. By incorporating valuable biological and chemical knowledge, kernels provide an efficient way to improve the accuracy of learning algorithms.

This work explores the use of learning algorithms to design and enhance the pharmaceutical properties of compounds [12, 13]. By starting with a training set containing approximately 100 peptides with their corresponding validated bioactivity (binding affinity, IC_50_, etc), we expect that a state-of-the-art kernel method will give a bioactivity model which is sufficiently accurate to find new peptides with activities higher than the 100 used to learn the model. This is possible because each peptide that possesses a small binding affinity contains information about subsequences of residues that can bind to the target. Learning a model can accelerate, but not solve, this costly process. *In-silico* predictions are faster and cheaper than *in-vitro* assays, however, predicting the bioactivity of all possible peptide to select the most bioactive ones would require a prohibitive amount of computational time. Indeed, this transforms the combinatorial drug discovery problem into an equally hard computational task.

We demonstrate that for a large class of kernel based models, it is possible to design an efficient algorithm guaranteed to find the peptide of maximal predicted bioactivity. This algorithm makes use of graph theory and recent work [14] on the prediction of the bioactivity and the binding affinity between peptides and a target protein. This algorithm can be part of an iterative combinatorial chemistry procedure that could speed up the discovery and the validation of peptide leads. Moreover, the proposed approach can be employed without known ligands for the target protein because it can leverage recent multi-target machine learning predictors [10, 14] where ligands for similar targets can serve as an initial training set. Finally, we demonstrate the effectiveness and validate our approach *in vitro* by providing an example of how antimicrobial peptides with proven activity were designed.

## Methods

### The Generic String kernel

String kernels are symmetric positive semi-definite similarity functions between strings. In our context, strings are sequences of amino acids. Such kernels have been widely used in applications of machine learning to biology. For example, the local-alignment kernel [15], closely related to the well-known Smith-Waterman alignment algorithm, was used for protein homology detection. It was however observed that kernels for large molecules such as proteins were not suitable for smaller amino acid sequences such as peptides [14]. Indeed, the idea of gaps in the local-alignment kernel or in the Smith-Waterman algorithm is well suited for protein homology, but a gap of only a few amino acids in a peptide would have important consequences on its ability to bind with a target protein. Many recently proposed string kernels have emerged from the original idea of the spectrum kernel [16] where each string is represented by the set of all its constituent *k*-mers. For example, the string *PALI* can be represented by the set of 2-mers {*PA, AL, LI*}. As defined by the *k*-spectrum kernel, the similarity score between two strings is simply the number of *k*-mers that they have in common. For example, the 2-spectrum similarity between *PALI* and *LIPAT* is 2, because they have two 2-mers in common (*PA* and *LI*).

To characterize the similarity between peptides, two different *k*-mer criteria were found to be important. First, two *k*-mers should only contribute to the similarity if they are in similar positions in the two peptides [17]. Second, the two *k*-mers should share common physico-chemical properties [18].

Meinicke and colleagues [17] proposed to weight the contribution of identical *k*-mers with a term that decays exponentially with the distance between their positions. If *i* and *j* denote the positions of the *k*-mers in their respective strings, the contribution to the similarity is given by

$$\exp\left(\frac{-{\left(i-j\right)}^{2}}{2\sigma_{p}^{2}}\right),$$ (1)

where *σ*_*p*_ is a parameter that controls the length of the decay.

Toussaint and colleagues [18] proposed to consider properties of amino acids when comparing similar *k*-mers. This was motivated by the fact that amino acids with similar physico-chemical properties can be substituted in a peptide while maintaining the binding characteristics. To capture the physicochemical properties of amino acids, they proposed to use an encoding function $\psi :𝓐\to {\mathbb{R}}^{d}$ where ***ψ***(*a*) = (***ψ***_1_(*a*), ***ψ***_2_(*a*), …, ***ψ***_*d*_(*a*)), to map every amino acid a∈𝓐 to a vector where each component ***ψ***_*i*_(*a*) encodes one of the *d* properties of amino acid *a*. In a similar way, we can define $\psi^{k}:{𝓐}^{k}\to {\mathbb{R}}^{dk}$ as an encoding function for *k*-mers, where

$$\psi^{k}(a_{1},a_{2},\ldots ,a_{k})\overset{\text{def}}{=}(\psi (a_{1}),\psi (a_{2}),\ldots ,\psi (a_{k})),$$ (2)

by concatenating *k* physico-chemical property vectors, each having *d* components. Throughout this study, the BLOSUM62 matrix was used in such a way that ***ψ***(*a*) is the line associated to the amino acid *a* in the matrix. It is now possible to weight the contribution of any two *k*-mers *a*_1_, …, *a*_*k*_ and ${a^{\prime }}_{1},\ldots ,{a^{\prime }}_{k}$ according to their properties:

$$\exp\left(\frac{-\;\|\psi^{k}(a_{1},\ldots ,a_{k})-\;\psi^{k}{\left({a^{\prime }}_{1},\ldots ,{a^{\prime }}_{k})\right\|}^{2}}{2\sigma_{c}^{2}}\right),$$ (3)

where ‖∙‖ denotes the Euclidean distance.

More recently, the Generic String (GS) kernel was proposed for small biological sequences and pseudo-sequences of binding interfaces [14]. The GS kernel similarity between an arbitrary pair (**x**, **x**′) of biological sequences is defined to be

$$GS(x,x^{\prime },k,\sigma_{p},\sigma_{c})\overset{\text{def}}{=}\sum_{l=1}^{k}\sum_{i=0}^{|x|-l}\sum_{j=0}^{|x^{\prime }|-l}\exp\left(\frac{-{\left(i-j\right)}^{2}}{2\sigma_{p}^{2}}\right)\exp\left(\frac{-\;\|\psi^{l}(x_{i+1},..,x_{i+l})-\psi^{l}{\left({x^{\prime }}_{j+1},..,x_{j+l})\right\|}^{2}}{2\sigma_{c}^{2}}\right).$$ (4)

Hence, the GS similarity between strings **x** and **x**′, is given by comparing their 1-mer, 2-mers, …up to their *k*-mers, with the position penalizing term of Equation (1) and the physico-chemical contribution term of Equation (3). The hyper-parameters *k*, *σ*_*p*_, *σ*_*c*_ are chosen by cross-validation.

This GS kernel is very versatile since, depending on the chosen hyper-parameters, it can be specialized to eight known kernels [14]: the Hamming kernel, the Dirac delta, the Blended Spectrum [8], the Radial Basis Function (RBF), the Blended Spectrum RBF [18], the Oligo [17], the Weighted degree [19], and the Weighted degree RBF [18]. It thus follows that the proposed method, based on the GS kernel, is also valid for all of these kernels.

Recently [14], the GS kernel was used to learn a predictor capable of predicting, with reasonable accuracy, the binding affinity of any peptide to any protein on the PepX database. The GS kernel has also outperformed current state-of-the-art methods for predicting peptide-protein binding affinities on single-target and pan-specific Major Histocompatibility Complex (MHC) class II benchmark datasets and three Quantitative Structure Affinity Model benchmark datasets. The GS kernel was also part of a method that won the 2012 Machine Learning Competition in Immunology [20]. External validation showed that the SVM classifier with the GS kernel was the overall best method to identify, given unpublished experimental data, new peptides naturally processed by the MHC Class I pathway. The proven effectiveness of this kernel made it ideal to tackle the present problem.

### The machine learning approach

In the binary classification setting, the learning task is to predict whether a peptide has a specific property such as binding to a target molecule. In the regression setting, the learning task is to predict a real value that quantifies the quality of a peptide, for example, its bioactivity, inhibitory concentration, binding affinity, or bioavailability. In contrast to classification and regression, the task we consider here (described in the next section) is ultimately to predict a string of amino acids.

In this paper, each learning example ((**x**, **y**), *e*) consists of a peptide **x**, a drug target **y**, which is typically a protein (but other biomolecules could be considered), and a real number *e* representing the bioactivity of the peptide **x** with the target **y**. In classification, *e* ∈ {+1, −1} denotes whether (**x**, **y**) has the desired property or not. Since predicting real values is strictly more general than predicting binary values, we focused on the more general case of real-valued predictors. Those learning examples are obtained from *in vitro* or *in vivo* experiments. The learning task is therefore to infer the value of *e* given new examples (**x**, **y**) that would not have been tested through experiments.

A predictor is a function *h* that returns an output *h*(**x**, **y**) when given any input (**x**, **y**). In our setting, the output *h*(**x**, **y**) is a real number that estimates the “true” bioactivity *e* between **x** and **y**. Such a predictor is said to be *multi-target* since its output depends on the ligand **x** and the target **y**. A multi-target predictor is generally obtained by learning from numerous peptides, binding to various proteins, for example, a protein family. For this reason, it can predict the bioactivity of any peptide with any protein of the family even if some proteins are not present in the training data [10, 14].

In contrast, a predictor *h*_**y**_(**x**) is said to be *target-specific* when it is dedicated to predict the bioactivity of any peptide **x** with a specific protein **y**. A target-specific predictor is obtained by learning only from peptides binding to a specific protein or from a multi-target predictor [10, 14]. For simplicity, we will focus on target-specific predictor but let us demonstrate how a target-specific predictor is obtained from a multi-target one.

Given a training set {((**x**_1_, **y**_1_), *e*_1_), …, ((**x**_*m*_, **y**_*m*_), *e*_*m*_)}, a large class of learning algorithms produce multi-target predictors *h* with the output *h*(**x**, **y**) on an arbitrary example (**x**, **y**) given by

$$h(x,y)=\sum_{q=1}^{m}\alpha_{q}k_{𝓨}(y,y_{q})k_{𝓧}(x,x_{q}),$$ (5)

where $k_{𝓨}:𝓨\times 𝓨\to \mathbb{R}$ and $k_{𝓧}:𝓧\times 𝓧\to \mathbb{R}$ are, respectively, the kernel functions between proteins and peptides, and *α*_*q*_ is the weight on the *q*-th training example. Since we use the GS kernel for $k_{𝓧}$, we obtain the target-specific predictor

$$h_{y}(x)=\sum_{q=1}^{m}\beta_{q}(y)GS(x,x_{q},k,\sigma_{p},\sigma_{c}).$$ (6)

Here the weight on the *q*-th training example is now given by *β*_*q*_(**y**). To obtain *h*_**y**_ from a multi-target predictor, we use $\beta_{q}(y)=\alpha_{q}k_{𝓨}(y,y_{q})$. When *h*_**y**_ is target-specific predictor learned only with peptides binding to **y**, we simply use *β*_*q*_(**y**) = *α*_*q*_. The remainder of this manuscript will focus on target-specific predictor in the form of Equation 6. This makes the proposed solution compatible for both target-specific and multi-target predictors. Also, since the weights on examples are given by *β*(**y**), we will see that the approach is valid regardless of the choice of kernel for the target protein.

The weight vector $\alpha \overset{\text{def}}{=}\;(\alpha_{1},\ldots ,\alpha_{m})$ depends on the learning algorithm used, but many algorithms produce prediction functions given by Equation (5), including the Support Vector Machine, the Support Vector Regression, the Ridge Regression, and Gaussian Processes. Note that all these learning methods require both kernels to be symmetric and positive semi-definite. This is the case for the GS kernel. The proposed solution for drug design is thus compatible with these popular bioinformatics learning algorithms [21]. However, some machine learning methods such as neural networks and its derivatives (deep neural networks) are not compatible with the proposed methodology.

For the sake of comparison, we would like to highlight that when *β*_*q*_(**y**) = 1/*m*, *k* = 1, *σ*_*p*_ = 0, and *σ*_*c*_ = 0 the predictor *h*_**y**_(**x**) in Equation (6) reduces to predict the probability of sequence **x** given the position-specific weight matrix (PSWM) obtained from the training set. Since *β*_*q*_(**y**), *k*, *σ*_*p*_, and *σ*_*c*_ can be arbitrary, the class of predictors we consider here is much more general.

Indeed, a PSWM consists of a position frequency matrix M:|𝓐|×l where *M*_*i, j*_ denotes the frequency of the *i*-th amino acid at the *j*-th position of peptides in the dataset. Since a PSWM assumes statistical independence between positions in the pattern, the probability that a sequence belongs to a certain pattern is given by summing the corresponding entries in *M*. PSWM are simple but have, however, been surpassed by modern machine learning algorithms [22, 23] since they assume independence between positions in the pattern. Moreover, they do not take into account the quantified bioactivity nor the similarities between amino acids. In addition, they require peptides to be aligned or of the same length. The method we present here have none of these serious limitations by allowing more sophisticated predictors to be learned.

### The combinatorial search problem

The main motivation for learning a predictor from training data is that, once an accurate predictor is obtained, finding druggable peptides would be greatly facilitated. It is true that replacing or reducing the number of expensive laboratory experiments by an *in silico* prediction will reduce costs. However, peptides having a low bioactivity do not qualify as drug precursors. Instead, we should focus on identifying the most bioactive ones. The computational problem is thus to identify and sort peptides according to a specific biological function. Let 𝓐 be the set of all amino acids, and ${𝓐}^{l}$ be the set of all possible peptides of length *l*. Then, finding the peptide $x^{⋆}\in {𝓐}^{l}$ that, according to *h*_**y**_, has the maximal bioactivity with **y**, amounts at solving

$$x_{y}^{⋆}=\arg\;\underset{x\in {𝓐}^{l}}{\text{max}}\;h_{y}(x).$$ (7)

This combinatorial problem is complex because, according to the predictor *h*_**y**_, the contribution of an amino acid at a certain position also depend on the *k* − 1 adjacent amino acids. This is the case since string kernel use *k*-mers to compare sequences. For that reason, each amino acid of the peptide cannot be optimized independently, but globally. Moreover, since the number of possible peptides grows exponentially with *l* (the length of the peptide), a brute force algorithm has an intractable complexity of $𝓞(|𝓐{|}^{l}∙\;𝓞(h_{y}))$ where $𝓞(h_{y})$ denotes the worst case time complexity for computing *h*_**y**_(**x**), the output of the predictor on peptide a **x**. Such an algorithm becomes impractical for any peptide exceeding 6 amino acids.

When facing such task, heuristics and stochastic optimization methods were generally the methods of choice [24, 25]. However, these methods often require prohibitive CPU time and are not guaranteed to find the optimal solution. In addition, these approaches are not capable of sorting the best solutions since they are designed to find a single maximum.

In the next section, we present an efficient algorithm guaranteed to solve Equation (7). We also present a second algorithm capable of sorting in decreasing order the peptides maximizing Equation (7). Both algorithms have low asymptotic computational complexity, yielding tractable applications for the design and screening of peptides.

### Finding the peptide of maximal bioactivity

**Figure 1:**
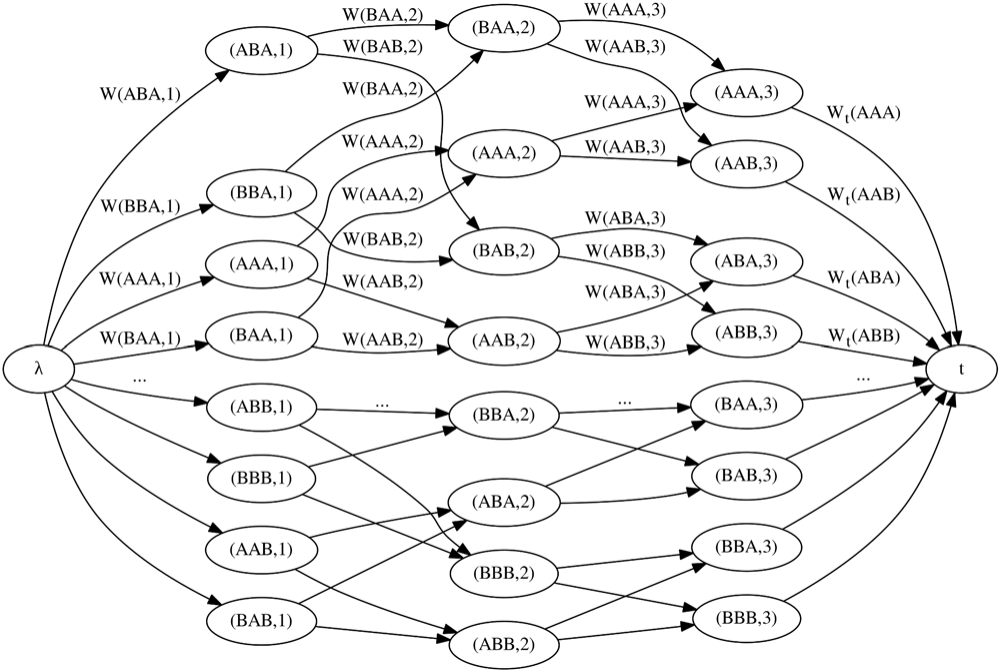
Illustration of the 3-partite graph *G* ^*h*_**y**_^ with *k* = 3 and a two letters alphabet 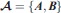. In this graph, every source-sink path represent a peptide of size 5 (*l* = *n* + *k* − 1) based on the alphabet {*A, B*}.

Here, we assume that we have, for a fixed target **y**, a prediction function *h*_**y**_(**x**) given by Equation (6). In this case, we show how the problem of finding, the peptide $x_{y}^{⋆}\in {𝓐}^{l}$ of maximal bioactivity reduces to the problem of finding the longest path in a directed acyclic graph (DAG). Note that, throughout this manuscript, we will assume that the length of a path is given by the sum of the weights on its edges. To solve this problem, we construct a DAG with a source and a sink vertex such that for all possible peptides $x\in {𝓐}^{l}$, there exists only one path associated to **x** that goes from the source to the sink. Moreover, the length of the path associated to **x** is exactly *h*_**y**_(**x**). Thus, if the size of the constructed graph is polynomial in *l*, any algorithm that efficiently solves the longest path problem in a DAG will also efficiently find the peptide of maximal bioactivity. A simplification of the graph is shown in Fig. 1 to assist in the comprehension of the formal definition that follows.

A directed bipartite graph is a graph whose vertices can be divided into two disjoint sets such that every directed edge connects a vertex of the first set to the second set. The construction of the graph will proceed as follows.

Let *k* be the maximal length of *k*-mers considered by the GS kernel. Let $U_{i}\overset{\text{def}}{=}{𝓐}^{k}\times \left\{i\right\}$, in other words, the set *U*_*i*_ contains all tuples (*s, i*) where *s* is a *k*-mer and *i* an integer. Let *G*_*i*_ = ((*U*_*i*_, *U*_*i*+1_), *E*_*i*_) be the *i*-th directed bipartite graph of some set where the set of directed edges *E*_*i*_ is defined as follows. Similarly as in the de Bruijn graph, there is a directed edge ((*s, i*), (*s*′, *i*+1)) from (*s, i*) in *U*_*i*_ to (*s*′, *i*+1) in *U*_*i*+1_ if and only if the last *k* − 1 amino acids of *s* are the same as the first *k* − 1 amino acids of *s*′. For example, in the graph of Fig. 1, there is an edge from (*ABA*, 1) to (*BAA*, 2) with *k* = 3. Note that ∀*i* ∈ ℕ, directed edges in *G*_*i*_ only go from vertices in *U*_*i*_ to vertices in *U*_*i*+1_. There are exactly |𝓐| edges that leave each vertex in *U*_*i*_ and there are exactly |𝓐| edges that point to each vertex in *U*_*i*+1_. Moreover, for any chosen integer *k*, $\left|U_{i}|\;=\;|U_{i+1}|\;=\;|{𝓐}^{k}\right|$ and $\left|E_{i}|\;=\;|{𝓐}^{k+1}\right|$. Note that there is a one-to-one correspondence between a sequence in ${𝓐}^{k+1}$ and a single edge path from a vertex in *U*_*i*_ to a vertex in *U*_*i*+1_.

We define a *n*-partite graph as the union of *n* − 1 bipartite graphs:

$$G_{1}\cup \ldots \cup G_{n-1}\overset{\text{def}}{=}((U_{1},U_{2},\ldots ,U_{n-1},U_{n}),E_{1}\cup \ldots \cup E_{n-1}).$$

Finally, let *G* ^*h*_**y**_^ be a *n*-partite graph with the addition of a source node *λ* and a sink node *t*. We choose the letter *λ* for the source node since it can be interpreted as the empty string (a 0-mer) node. There is a directed edge from *λ* to all nodes of *U*_1_ and from all nodes of *U*_*n*_ to *t*. For example, the graph illustrated in Fig. 1 is a 3-partite graph with a source and a sink node when the *k*-mer are of size 3 and the alphabet has two letters: A and B.

Throughout this manuscript, we will only focus on paths starting at *λ*, the source node, and ending at *t*, the sink node. For this reason, by choosing *n* = *l* −*k* + 1 we obtain the one-to-one correspondence between each peptide of ${𝓐}^{l}$ and each path *λ*, *u*_1_, …, *u*_*n*_, *t* where *u*_*i*_ ∈ *U*_*i*_. For example, in Fig. 1 the peptide ABAAA of size *l* = 5 is represented by the path *λ*, (*ABA*, 1), (*BAA*, 2), (*AAA*, 3), *t*.

Let us now describe how edges in *G* ^*h*_**y**_^ are weighted in order for the length of a path associated to **x** to be exactly *h*_**y**_(**x**), the predicted bioactivity of **x**. Using the definition of the GS kernel, given at Equation (4), and the general class of predictors, given by Equation (6), we can rewrite *h*_**y**_(**x**) as

$$h_{y}(x)=\overset{m}{\underset{q=1}{\sum }}\;\beta_{q}(y)\overset{k}{\underset{p=1}{\sum }}\overset{|x|-p}{\underset{i=0}{\sum }}\overset{|x_{q}|-p}{\underset{j=0}{\sum }}\exp\left(\frac{-{\left(i-j\right)}^{2}}{2\sigma_{p}^{2}}\right)\exp\left(\frac{-\|\psi^{p}(x_{\left[i+1\right]},..,x_{\left[i+p\right]})-\psi^{p}{\left(x_{q}{}_{\left[j+1\right]},..,x_{q}{}_{\left[j+p\right]})\right\|}^{2}}{2\sigma_{c}^{2}}\right).$$

For any *k*-mers *s* and any *i* ∈ {1, …, *n*}, we define

$$W(s,i)\overset{\text{def}}{=}\overset{m}{\underset{q=1}{\sum }}\;\beta_{q}(y)\overset{k}{\underset{p=1}{\sum }}\overset{|x_{q}|-p}{\underset{j=0}{\sum }}\exp\left(\frac{-{\left((i-1)-j\right)}^{2}}{2\sigma_{p}^{2}}\right)\exp\left(\frac{-\|\psi^{p}(s_{1},\ldots ,s_{p})-\psi^{p}(x_{q}{}_{\left[j+1\right]},..,x_{q}{}_{\left[j+p\right]}){\|}^{2}}{2\sigma_{c}^{2}}\right)$$ (8)

as the weight on edges heading to the node $\left(s,i)\in {𝓐}^{k}\times \{1,\ldots ,n\right\}$. The function *W* weight all edges of *G* ^*h*_**y**_^ except those heading to the sink vertex *t*. When *k* > 1, edges ((*s, n*), *t*), heading to the sink vertex *t*, are weighted by the function

$$W_{t}(s)=\sum_{j=1}^{k-1}W(s_{j+1}\ldots s_{k},n+j),$$ (9)

otherwise, *W*_*t*_(*s*) = 0 when *k* = 1.

For *n* = *l* − *k* + 1, we now have that

$$h_{y}(x)=W_{t}(x_{n},..,x_{l})+\sum_{i=1}^{n}W(x_{i},..,x_{i+k-1},i).$$

Therefore, every path from the source to the sink in *G* ^*h*_**y**_^ represents a unique peptide $x\in {𝓐}^{l}$ and its estimated bioactivity *h*_**y**_(**x**) is given by the length of the path.

The problem of finding the peptide of highest predicted activity thus reduces to the problem of finding the longest path in *G* ^*h*_**y**_^. Despite being NP-hard in the general case, the longest path problem can be solved by dynamic programming in $𝓞(|V(G^{h_{y}})|+|E(G^{h_{y}})|$ for a directed acyclic graph, given a topological ordering of its vertices. By construction, *G* ^*h*_**y**_^ is clearly acyclic and its vertices can always be topologically ordered by visiting them in the following order: *λ*, *U*_1_, …, *U*_*n*_, *t*. Since *G* ^*h*_**y**_^ has $n|𝓐{|}^{k}+2$ vertices and $2|𝓐{|}^{k}+\;(n-1)|𝓐{|}^{k+1}$ edges, the complexity of the algorithm will be dominated by the number of edges. Therefore, the proposed algorithm has a complexity of $𝓞(n|𝓐{|}^{k+1})$. Recall that *k* is a constant and *l* is the length of the best peptide we are trying to identify. Thus, *n* must be equal to *l* −*k* + 1.

Note that Equation (8) has to be evaluated for each edge of the graph. The dynamic programming algorithm proposed for the computation of the GS kernel [14] can easily be adapted to efficiently evaluate this equation. In that case, the complexity of the weight function is reduced to 𝓞(m∙l∙k).

Small values of *k* are motivated by the fact that $\|\;\psi^{k}(a_{1},..,a_{k})\;-\;\psi^{k}({a^{\prime }}_{1},..,{a^{\prime }}_{k})\;\;{\|}^{2}$ is a monotonically increasing function of *k*. Equation (3) will thus vanish exponentially fast as *k* increases. Long *k*-mers will thus have negligible influence on the estimated bioactivity, explaining why small values of *k* ≤ 6 ≪ *l* are empirically chosen by cross-validation. Therefore, the time complexity of the proposed algorithm is orders of magnitude lower than the brute force algorithm which is in $𝓞(|𝓐{|}^{l})$ since *k* ≤ 6 ≪ *l* in practice. The pseudo-code to find the longest path in *G* ^*h*_**y**_^ is given in Box 1.

**Box 1. Algorithm for finding the longest path between the source node *λ* and the sink node *t* in *G* ^*h*_y_^**

*length*_*to* = array with $n|𝓐{|}^{k}+\;2$ entries initialized to −∞

*predecessor* = array with $n|𝓐{|}^{k}+\;2$ entries

**for all** $s\in {𝓐}^{k}$ **do**        ▷**Edges leaving the source node**

  
*length*_*to*[*s*, 1] ← *W*(*s*,1)

**end for**

**for** *i* = 2 → *n* **do**        ▷**Edges from the core of** *G* ^*h*_**y**_^

 
**for all** $s\in {𝓐}^{k}$ **do**

  
**for all** a∈𝓐 **do**

   
*s*′ ← *s*_2_, …, *s*_*k*_, *a*        ▷**Note that *s*′ is a *k*-mers**

   
**If** *length*_*to*[*s*′, *i*] ≤*length*_*to*[*s*, *i* −1] + *W*(*s*′, *i*) **then**

    
*length*_*to*[*s*′, *i*] ← *length*_*to*[*s*, *i* − 1] + *W*(*s*′, *i*)

    
*predecessor*[*s*′, *i*] ← *s*

   
**end if**

  
**end for**

 
**end for**

**end for**

*max*_*length* ← − ∞

*longest*_*path* ← *λ*

**for all** $s\in {𝓐}^{k}$ **do**        ▷**Edges heading to the sink node**

 
**If** *max*_*length* ≤ *length*_*to*[*s*, *n*] + *W*_*t*_(*s*) **then**

  
*max*_*length* ← *length*_*to*[*s*, *n*] + *W*_*t*_(*s*)

  
*longest*_*path* ← *s*

 
**end if**

**end for**

**for** *i* = *n* → 2 **do**        ▷**Backtrack using the predecessors**

 
*s*_1_, …, *s*_*k*_ ← *predecessor*[*longest*_*path*_[1:*k*]_, *i*]

 
*longest*_*path* ← *s*_1_, *longest*_*path*

**end for**

**return** *longest*_*path*

### Finding the *K* peptides of maximal bioactivity

In the previous section, we demonstrated how the problem of finding the peptide of greatest predicted bioactivity was reduced to the problem of finding a path of maximal length in the graph *G* ^*h*_**y**_^. By using the same arguments, finding the peptide with the second greatest predicted activity reduces to the problem of finding the second longest path in *G* ^*h*_**y**_^. By induction, it follows that the problem of finding the *K* peptides of maximal predicted activity reduces to the problem of finding the *K*-longest paths in *G* ^*h*_**y**_^. The closely-related *K*-shortest paths problem has been studied since 1957 and attracted considerable attention following the work of Yen [26]. Yen’s algorithm was later improved by Lawler [27]. Both algorithms make use of a shortest path algorithms to solve the *K*-shortest paths problem. By exploiting some restrictive properties of *G* ^*h*_**y**_^, Yen’s algorithm for the *K*-shortest paths was adapted, shown in Box 2, to find the *K*-longest paths in *G* ^*h*_**y**_^. It uses a variant of the longest path algorithm presented in the previous section, that allows a path to start from any node of the graph. Lawler improvement to the algorithm is not part of the presented algorithm to avoid unnecessary confusion but is part of the implementation we provide. The time complexity of the resulting algorithm is competitive with the latest work on *K*-shortest paths algorithms [28, 29].

**Box 2 Algorithm for finding the *K*-longest paths in *G* ^*h*_y_^**

*A* = **array with** *K* **entries initialized with the empty string**

*B* = **max-heap to store potential paths and their lengths**

*A*[0] ← **LongestPath** (*G* ^h_**y**_^, λ, *t*)

**for** *i* = 0 → *K* − 1 **do**

 
**for all** (*a, j*) ∈ (λ, (*A* [*i*]_[0:*k*]_, 1), …, (*A* [*i*]_[*l* − *k*:*l*]_, *n*)) **do**        ▷**Nodes of the previous path**

  (*V, E*) ← *G* ^h_**y**_^

  
**root** ← *A*[*i*]_[0:*j*+*k*]_

  
**for** *r* = 0 → *i* **do**

   
**If** *A*[*r*]_[0:*j*+*k*]_ = *root* **then**

    
*E* ← *E*\(*A*[*r*]_[*j*:*j*+*k*]_, *j*)

   
**end if**

  
**end for**

  
*x* ← **root** + **LongestPath**((*V*, *E*), (*a*, *j*), *t*)

  
**if** *x* ∉ *B* ∪ *A* **then**

   
*B.push*(*x*, *h*_*y*_(*x*))        ▷**Add the string and its length to the max-heap**

  
**end if**

 
**end for**

 
*A*[*i* + 1] ← B.pop()        ▷***B*’s longest path becomes the *i*-th longest path**

**end for**

**return A**

The algorithm of Box 2 was implemented in a combination of both C and Python, the source code is freely available at http://graal.ift.ulaval.ca/peptide-design/. To validate the implementation and prevent potential flaws, it was successfully used to exhaustively sort all possible peptides of length 1 to 5 with various values of *k, σ*_*p*_, and *σ*_*c*_.

Having the *K* best peptides sorted according to their predicted bioactivity will provide valuable information with the potential of accelerating functional peptide discovery. Indeed, the best peptide candidates can be synthesized by an automated peptide synthesizer and tested *in vitro*. Such a procedure will allow rapid *in vitro* feedback and minimize turnaround time. Also, in the next section, we will describe how the *K* best predicted peptides can be utilized to predict a binding motif for a new, unstudied protein. Such a motif should assist researchers in the early study of a target and for the design of peptidomimetic compounds by providing residue preferences.

### From *K*-longest paths to motif

It is easy to use the *K*-longest paths algorithm to predict a motif by simply loading the *K* peptides to an existing motif tool. In this case, the motif is a property of the learned model *h*_**y**_(**x**) as opposed to a consensus among known binding sequences. When *h*_**y**_(**x**) is obtained from a multi-target model *h*(**x**, **y**), it is then possible to predict affinities for proteins with no known ligand by exploiting similarities with related proteins. It is therefore feasible to predict a binding motif for a target with no known binders. To our knowledge, this has never been realized successfully.

### Protocol for split and pool peptide synthesis

Split and pool combinatorial peptide synthesis is a simple but efficient way to synthesize a very wide spectrum of peptide ligands. It has been used for the discovery of ligands for receptors [30, 31], for proteins [32–35] and for transcription factors [36, 37]. To synthesize several peptides of length *l* using the 20 natural amino acids, the standard approach is to use one reactor per natural amino acid and a pooling reactor. At every step of the experiment, all reactors are pooled into the pooling reactor which is then split, in equal proportions, back into the 20 amino acid reactors. Within this standard approach, each peptide in ${𝓐}^{l}$ has an equal probability of being synthesized. Since the number of polystyrene beads (used to anchor every peptide) is generally orders of magnitude smaller than $|𝓐{|}^{l}$, only a vanishingly small fraction of the peptides in ${𝓐}^{l}$ can be synthesized in each combinatorial experiment.

Clearly, not every peptide has an equal probability of binding to a target. More restrictive protocols have been proposed to increase the hit ratio of this combinatorial experiment. For example, one could fix certain amino acids at specific positions or limit the set of possible amino acids at this position (for example, only use hydrophobic amino acids). Such practice will impact the outcome of the combinatorial experiment. One can probably increase the hit ratio by modifying (wisely) the proportion of amino acids that can be found at different positions in the peptides. To explore more thoroughly this possibility, let us define a (combinatorial chemistry) *protocol* *P* by a *l*-tuple containing, for each position *i* in the peptide of length *l*, an independent distribution ${𝓟}_{i}(a)$ over the 20 amino acids a∈𝓐. Hence, we define a protocol *P* by

$$P\overset{\text{def}}{=}({𝓟}_{1},\ldots ,{𝓟}_{l}).$$ (10)

Consequently, the peptides produced by this protocol will be distributed following the joint distribution ${𝓟}_{1}\times \;\ldots \;\times {𝓟}_{l}$. Hence, the probability of synthesizing a peptide **x** of size *l* is given by

$$P(x)=\prod_{i=1}^{l}{𝓟}_{i}(x_{i}).$$ (11)

Note that *P* formally defines a position-specific weight matrix (PSWM) that can be illustrated as a motif. Moreover, this family of protocols is easy to implement in the laboratory since, at each step *i*, it only requires splitting the content of the pooling reactor in proportions equal to the distribution ${𝓟}_{i}$ over amino acids. For example, if at position *i*, we wish to sample uniformly over each amino acid, then we will use ${𝓟}_{i}(a)=1/20$ for all a∈𝓐. If on the other hand, we wish, at position *i*, to sample amino acids C, D, or E with equal probability and the rest of the amino acids with probability 0, then we use ${𝓟}_{i}(a)=1/3$ for *a* ∈ {*C, D, E*} and ${𝓟}_{i}(a)\;=\;0$ for *a* different from either *C*, *D*, or *E*.

### Expected outcome of a library given a protocol

We present a method for efficiently computing exact statistics on the screening outcome of a peptide library synthesized according to a protocol *P*. Specifically, we present an algorithm to compute the average predicted bioactivity and its variance over all peptides that a protocol can synthesize. Note that it is intractable to compute these statistics by predicting the activity of each peptide.

Such statistics will, for example, assist chemists in designing a protocol with a greater hit ratio and avoid superfluous experiments. Furthermore, we will demonstrate in the next section that the computation of these statistics can be part of an iterative procedure to accelerate the discovery of bioactive peptides. Indeed, having the average predicted bioactivity data will help with the design of a protocol that synthesizes as many potential active candidates as possible. In addition, the predicted bioactivity variance will allow for better control of the exploration/exploitation trade off of the experiment. Finally, as described in the previous section, a widely used practice for optimizing peptides is to assign residues at certain positions or restrict them to those that have specific properties such as charge or hydrophobicity. It is now possible to quantify how such procedure will impact the bioactivity of combinatorially synthesized peptides.

The proposed approach makes use of the graph *G* ^*h*_**y**_^, the protocol *P*, and a dynamic programming algorithm that exploits recurrences in the factorization of first and second order polynomials. This allows for the efficient computation of the first and second moment of *h*_**y**_ when peptides are drawn according to the distribution *P*. Then, the average and variance can easily be obtained from the first two moments. Details of the approach and the algorithm are given in supplementary material (see S1 Text).

### Application in combinatorial drug discovery

**Figure 2:**
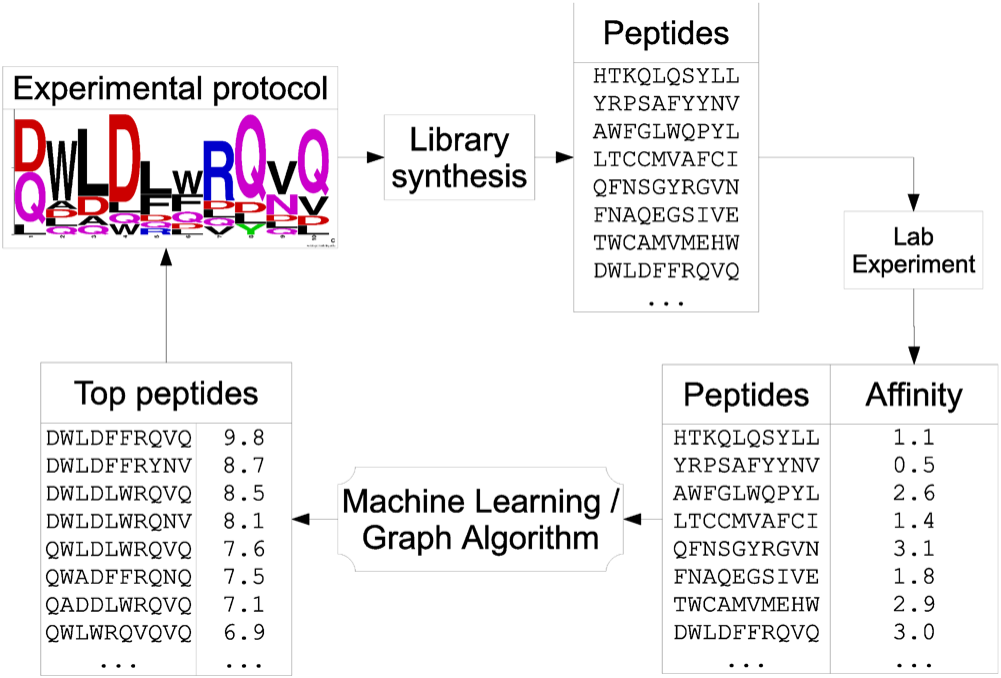
Iterative process for the design of peptide ligands.

We propose an iterative process that makes use of the proposed algorithms to accelerate the discovery of bioactive peptides. The procedure is illustrated in Fig. 2. First, an initial set of random peptides is synthesized, typically using a split and pool approach. The peptides are assayed in laboratory to measure their bioactivities. At this point, most peptides are poor candidates. They are then used as a training set to produce a predictor *h*_**y**_. Next, *h*_**y**_ is used for the generation of *K* bioactive peptides by finding the *K*-longest paths in *G* ^*h*_**y**_^ as described previously. A protocol *P* is constructed from these *K* bioactive peptides to assist the next round of combinatorial chemistry. Then, the algorithm described in the previous section is used to predict statistics on the protocol *P*. This ensures that the protocol meets expectations in terms of quality (average predicted bioactivity) and diversity (predicted bioactivity variance). To lower costs, one should proceed to synthesize and test the library only if expectations are met. This process can be repeated until the desired bioactivity is achieved.

## Results/ Discussion

### Data

Two public datasets were used to test and validate our approach. The first dataset consisted of 101 cationic antimicrobial pentadecapeptides (CAMPs) from the synthetic antibiotic peptides database [38]. Peptide antibacterial activities are expressed as the logarithm of bactericidal potency which is the average potency over 24 bacteria such as *Escherichia coli, Bacteroïdes fragilis*, and *Staphylococcus aureus*. The average antibacterial activity of the CAMPs dataset was 0.39 and the best peptide had an activity of 0.824.

The second dataset consisted of 31 bradykinin-potentiating pentapeptides (BPPs) reported in [39]. The bioactivities are expressed as the logarithm of the relative activity index compared to the peptide VESSK. The average bioactivity of the BPPs dataset was 0.71 and the best peptide had an activity of 2.73.

### Improving the bioactivity of peptides

To assess the capability of the proposed approach to improve upon known peptides, two experiments were carried out using the CAMPs and BPPs peptide datasets. For both experiments, a predictor of biological activity was learned by kernel ridge regression (KRR) for the each datasets: *h*_*CAMP*_ and *h*_*BPP*_. Hyper-parameters for the GS kernel (*k, σ*_*c*_, *σ*_*p*_) and the kernel ridge regression (*λ*) were chosen by standard cross-validation: *k* = 2, *σ*_*c*_ = 6.4, *σ*_*p*_ = 0.8, and *λ* = 6.4 for *h*_*CAMP*_ and *k* = 3, *σ*_*c*_ = 0.8, *σ*_*p*_ = 0.2, and *λ* = 0.4 for *h*_*BPP*_.

#### *In silico* validation

Using the *K*-longest path algorithm and the learned predictors, we generated the *K* peptides (of the same length as those of the training data) having the greatest predicted biological activity.

**Figure 3:**
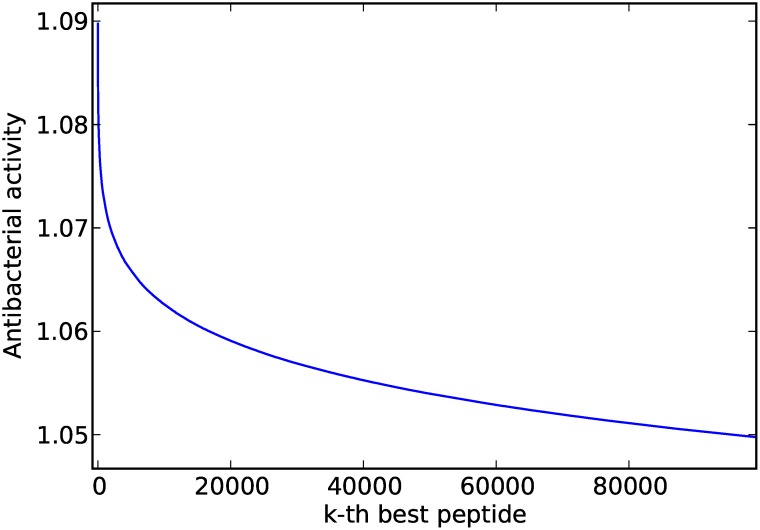
The 100,000 peptides with highest antimicrobial activity found by the *K*-longest path algorithm.

For the CAMPs dataset, the proposed approach predicted that peptide WWKWWKRLRRLFLLV should have an antibacterial potency of 1.09, a logarithmic improvement of 0.266 over the best peptide in the training set (GWRLIKKILRVFKGL, 0.824), and a substantial improvement over the average potency of that dataset (average of 0.39). The antimicrobial activity of the top 100,000 peptides are showed in Fig. 3. We observe a smooth power law with only a few peptides having outstanding biological activity, as expected. As we will see in the next section, peptides at the top of the curve, hence having the best bioactivities, are very unlikely to be found by chance.

On the BPPs dataset, the proposed approach predicted that the pentapeptide IEWAK should have an activity of 2.195, slightly less than the best peptide of the training set (VEWAK, 2.73, predicted as 2.192). However, the predicted activity of IEWAK is much better than the average peptide activity of the dataset, which is 0.71. One may ask why IEWAK has a lower predicted biological activity than VEWAK, which was part of the training data. It is common for machine learning algorithms to sacrifice accuracy on the training data to prevent overfitting. Despite this small discrepancy, the model is very accurate on the training data (correlation coefficient of 0.97). Another possible explanation for this discrepancy is that the biological activity of VEWAK could be slightly erroneous as the learning algorithm could not find a simple model given such an outlier. It seems that the predicted activity of VEWAK is more coherent with the whole data than its measured activity.

Hence, our proposed learning algorithm predicts new peptides having biological activities equivalent to the best of the training set and, in some cases, substantially improved activities. The next section present an *in vitro* experiment that clearly demonstrate that in a real world test, our approach can generate bioactive peptides.

#### *In vitro* validation

To further validate the approach, a number of antimicrobial peptides identified during the *in silico* validation were synthesized. Their antimicrobial activity against *Escherichia coli* and *Staphylococcus aureus* were measured in a growth inhibitory assay. Details on the synthesis and assay are given in the supplementary material (see S1 Text). The peptides were obtained using *h*_*CAMP*_, the same predictor used during the previous validation.

The two most active peptides of the CAMPs dataset (Peptide #5 and #6) were synthesized for comparison. We also synthesized one peptide with poor activity (Peptides #7) as a control. We used the proposed approach with the predictor *h*_*CAMP*_ to generate a list of *K* = 1,000 peptide candidates with the highest predicted activity. From this list, we greedily selected three peptides such that they all differed by at least 4 amino acids from each others. This was done to maximize the chemical diversity among them. We then tested these peptides (Peptides #2, #3, #4) in a growth inhibitory assay. Results from the minimal inhibitory concentration assay are shown in Table 2. Two of the three candidates had activities equal to the best peptide of the CAMPs dataset. We were intrigued by the failure of Peptide #4 and after investigation, the weak activity was due to poor water solubility. In a second series, we ensured that a filter for water solubility was employed. In this second series of tests, Peptide #1 showed (at least against *E. coli*) better activity than any of the original candidates from the CAMPs dataset, demonstrating that, in this limited biological experiment, we could improve the putative candidates using the proposed machine learning methodology. Finally, all predicted antimicrobial peptides are significantly different from those of the training set, sharing only 40% similarity with their most similar peptide in the CAMPs dataset.

### Simulation of a drug discovery

Previously, we described a methodology (illustrated in Fig. 2) that uses machine learning to guide the combinatorial chemistry search for finding peptides with high bioactivity. However, before conducting such an expensive and time-consuming experiment, it is reasonable to first investigate, *in silico*, if the proposed methodology could find peptides having high bioactivity.

Hence, to validate the proposed methodology, we replaced the laboratory experiments that would quantify the bioactivity level of peptides by an oracle for each dataset. We choose to use *h*_*CAMP*_ and *h*_*BPP*_ as oracle as they represent, so far, the best understanding of the studied phenomena. These oracles will be used to quantify the bioactivity level of randomly generated peptides and those proposed by our methodology. Note that, examples used to learn the oracles are not available to our algorithm during the validation. Consequently, the validation method used was the following.

1. We randomly generated *R* peptides on a computer instead of using combinatorial chemistry.
2. To measure the bioactivities, we replaced the laboratory experiments by the oracle.
3. We used these random peptides of low bioactivities to learn a second predictor *h*_*random*_.
4. The predictor *h*_*random*_ is used to initiate the graph-based approach. We then obtained the *K* potentially best peptides.
5. The new peptides bioactivities are validated by the oracle (instead of performing laboratory experiments).
6. Finally, we compared the bioactivities of the initial set of peptides (randomly generated) and those proposed by our approach.

**Finding peptides with high bioactivity** The testing methodology was conducted twice on both the CAMPs and the BPPs datasets. Once by generating *R* = 100 peptides at Step 1 and considering the *K* = 100 best predicted peptides at Step 4 of the methodology, and then by starting over the validation with *R* = 1,000 and *K* = 1,000. Statistics on the random peptides and those proposed by our approach are shown in Table 1.

**Table 1:** Results from the drug discovery simulation.

		*R* Randomly Picked		*K* Best Predicted		*h*_*random*_	
Dataset	Value of *R* and *K*	Average	Max.	Average	Max.	Correlation Coef.
CAMPs	100	−0.58	0.17	0.76	0.83	0.51
	1000	−0.59	0.18	1.07	1.09	0.90
BPPs	100	0.31	1.39	1.50	2.04	0.67
	1000	0.26	1.36	1.66	2.20	0.93

Bioactivity comparison between the standard combinatorial screening (*R* random picked peptides) and the proposed approach (*K* best predicted peptides), initiated with the same *R* random peptides. Values are logarithm of bactericidal potencies. The correlation coefficients of *h*_*random*_ were computed using the oracle.

**Table 2:** *In-vitro* minimal inhibitory concentration assay.

	Predicted	MIC (μ*g*/*ml*)		Most Similar Peptide in the Training Set	
#	Peptide Sequence	*>E. coli*	*S. aureus*	Peptide Sequence	% Similarity
1	YWKKWKKLRRIFMLV	2	8	LWKLFKKIRRVLRVL	40.0
2	WWKRWKKLRRIFLML	4	4	LWKLFKKIRRVLRVL	40.0
3	WWKRWKRIRRIFMMV	4	8	LWKLFKKIRRVLRVL	40.0
4	WWKWWKRLRRLFLLV	16	16	LWKLFKKIRRLLKVL	46.6
5	KWKLFKGIRAVLKVL	4	8	-	-
6	GWRLIKKILRVFKGL	4	4	-	-
7	KWKLFLGILAVLKVL	> 32	> 32	-	-

Minimal inhibitory concentration (MIC) from ***in vitro*** CAMPs assay. We predicted peptides 1 to 4, peptides 5 to 7 are controls from the training set. The ordering of the peptides do not reflect their predicted bioactivities.

As expected, on both datasets, the number of peptides drawn (*R*) had no impact on the average activity of randomly drawn peptides. Also, on both datasets, increasing *R*, the number of random peptides, had no significant influence on the bioactivity of the best peptide found. This support the main hypothesis upon which this work is based, random peptides will consistently be of low activity. This also indicates that combinatorial chemistry alone does not allow one to find the best peptides. It requires hints to orient its search. The next paragraph points out that our machine learning approach can provide such hints.

Using the same *R* = 100 (low bioactivity) random peptides to initiate our method (i.e. train the predictor *h*_*random*_), we were able to reach an antimicrobial potency of 0.83 (according to oracle, not to the prediction of *h*_*random*_). Such antimicrobial potency is similar to the best peptide of the (unseen) CAMPs dataset and much better than the best of the *R* = 100 random peptides. By increasing to *R* to 1,000, we found a peptide having a potency of 1.09 according to the oracle. This peptide surpasses the best known peptide of the CAMPs dataset and is also far superior to the best of the *R* = 1,000 random peptides. On the BPPs dataset, the proposed approach also considerably outperformed the random approach on both the best peptide found and the average bioactivity. Finally, on both datasets, increasing the number of initial peptides from *R* = 100 to *R* = 1,000 was more beneficial on the bioactivity measures than the random approach.

**Comparing *h*_*random*_ and the oracle accuracies on the CAMPs and BPPs databases** To provide additional support for its accuracy, predictor *h*_*random*_ was used to predict the bioactivity values of unseen but *in-vitro* validated peptides of the CAMPs and BPPs databases. The Pearson correlation coefficient (PCC, also known as the Pearson’s *r*) was computed between *h*_*random*_ predictions and the values in both databases. Since, in this simulation, *h*_*random*_ was learned only with random peptides that, as pointed out above, have low bioactivity, it is interesting to evaluate its accuracy on these databases.

Correlation coefficients are shown in the last column of Table 1. When initiated with *R* = 1,000 random peptides, it achieves a correlation coefficient of 0.90 (CAMPs) and 0.93 (BPP). In comparison, the oracle achieved a correlation coefficient of 0.91 (CAMPs) and 0.97 (BPP) on the same peptides. These were however used to train the oracle. Given that *h*_*random*_ is bound to be less accurate than the oracle, these results demonstrate the capability of our approach to learn a predictor using low bioactivity peptides to obtain highly active ones.

**Figure 4:**
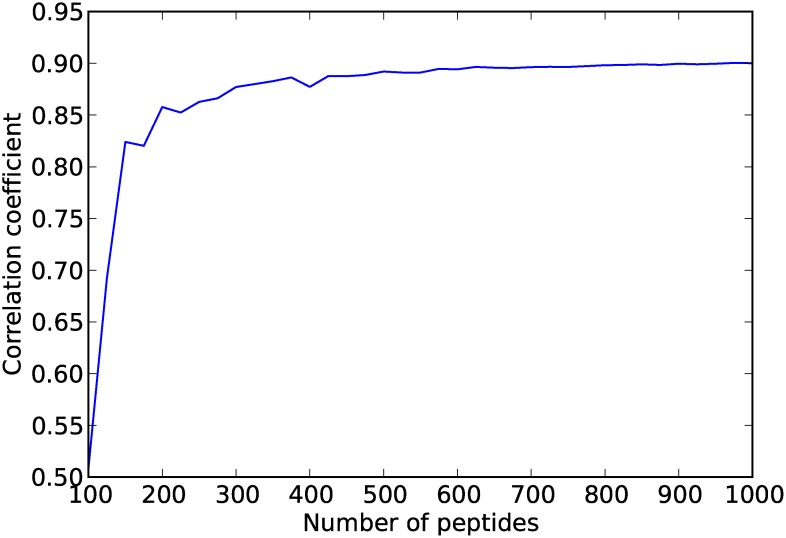
Correlation coefficient of *h*_*random*_ predictions on the CAMPs data while varying *R*, the number of random peptides used as training set.

Fig. 4 shows the correlation coefficient of *h*_*random*_ on the CAMPs data when varying R, the number of random peptides used for training. Near optimal accuracy is reached when *h*_*random*_ is initiated with approximately *R* = 300 peptides. This suggests that the proposed method can achieve excellent performance with a database of modest size.

### Binding motifs results and comparison with PSWM

The results presented here serve to demonstrate the ability of the proposed approach to predict potential functional motifs and to compare to position-specific weight matrix (PSWM) as they can be illustrated as a motif.

**Figure 5:**
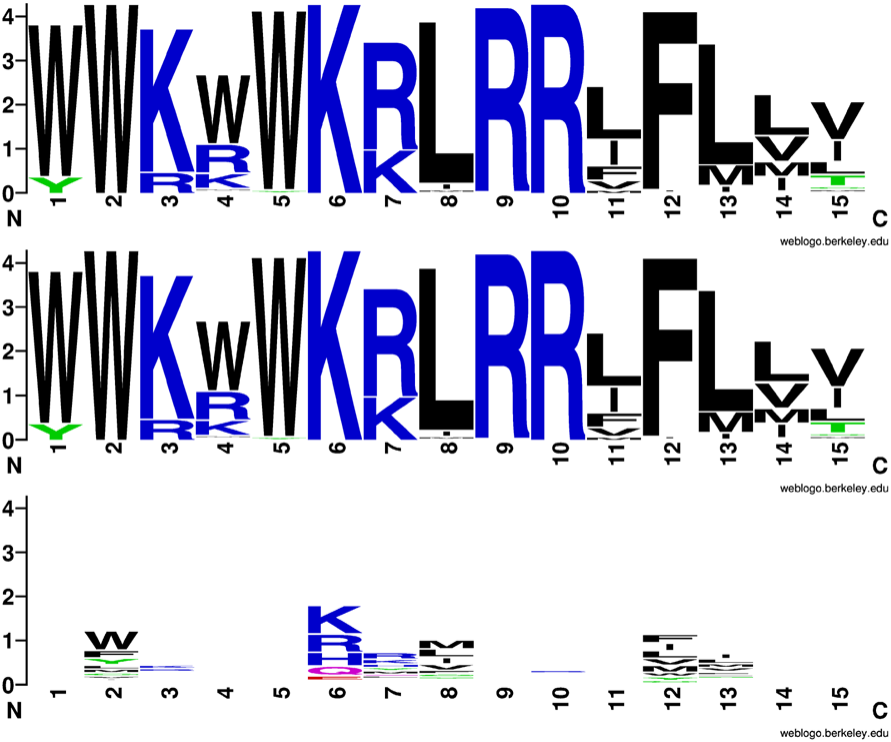
CAMP bioactivity motifs. Top motif: the best 1,000 peptides obtained from the oracle. Middle motif: the best 1,000 peptides obtained from *h*_*random*_. Bottom motif: the best 1,000 out of 1,000,000 random peptides.

For the CAMPs dataset, we used *h*_*CAMP*_ as oracle and hidden all peptides in this dataset from the rest of the procedure. Using the oracle, we predicted the best *K* = 1,000 peptides and generated a bioactivity motif using these candidates (top panel of Fig. 5). Our goal was to assess how much of that reference motif we could rediscover if we were to hide all the CAMPs dataset during the validation.

Using only the predictor *h*_*random*_, trained on *R* = 1,000 randomly generated peptides, we generated the motif representing the *K* = 1,000 best predicted peptides (according to *h*_*random*_). The motif is shown in middle panel of Fig. 5. We were able to recover all the reference motif signal using only weakly active peptides and *h*_*random*_. To push the analysis even further, we also computed the motif when *h*_*random*_ is trained with only *R* = 100 random peptides. Even then (motif not shown), for 12 of the 15 residue positions, we were able to correctly identify the dominant amino acid property (polar, neutral, basic, acidic, hydrophobic). This can be achieved since the GS kernel encodes amino acids physico-chemical properties.

This provides evidence that the proposed approach could uncover complex signals for new, poorly understood, proteins. For example, one could learn a multi-target predictor for peptides binding to the major histocompatibility complex [14]. Since these molecules are highly polymorphic, it would be interesting to predict antigen binding motifs for a specific segment of a population or even a single patient. This would have applications in the design of epitope based vaccines [40] and provide additional insight into autoimmune diseases.

To compare our approach to PSWM, we took the same *R* = 1,000 randomly picked peptides used to train the predictor *h*_*random*_ and generated a PSWM. The signal in PSWM motif was very poor, generating a meaningless motif (not shown). We increased the number of random peptides to *R* = 1,000,000 and only selected the best *K* = 1,000 to produce a PSWM whose motif is shown in the bottom panel of Fig. 5. Despite this big advantage, the motif of the PSWM shows minimal information.

This clearly illustrates the potential of the proposed approach for accelerating the discovery of potential peptidic effectors and, possibly, for achieving a better understanding of the binding mechanisms of polymorphic molecules.

## Conclusion and Outlook

We proposed an efficient graph-based algorithm to predict peptides with the highest biological activity for machine learning predictors using the GS kernel. Combined with a multi-target model, it can be used to predict binding motifs for targets with no known ligands.

To increase the hit ratio of combinatorial libraries, we demonstrated how a combinatorial chemistry protocol relates to a PSWM. This allowed us to compute the expected predicted bioactivity and its variance that can be exploited in combinatorial chemistry. These steps can be part of an iterative drug discovery process that will have immediate use in both the pharmaceutical industry and academia. This methodology will reduce costs and the time to obtain lead peptides as well as facilitating their optimization. Finally, the proposed approach was validated in a real world test for the discovery of new antimicrobial peptides. These *in vitro* experiments confirmed the effectiveness of the new peptides uncovered.

The *K*-best peptides were shown to be valuable for the design of split and pool libraries. However, in such libraries, it is unclear how we should prioritize high activity candidates (average) over the chemical diversity (variance). This exploration/exploitation trade-off warrants further investigation. The fast computation of the bioactivity average and variance given a combinatorial chemistry protocol will certainly help to exploit this trade-off. Moreover, the method could easily be adapted to optimize multiple objectives simultaneously, for example, the bioactivity at the expense of mammalian cell toxicity or bioavailability when such data are available. In addition, the method could be expanded to cyclic peptides and chemical entities commonly found in clinical compounds. Finally, this method shows great promise in immunology, where antigen binding motifs for unstudied major histocompatibility complexes could be uncovered using a multi-target predictor.

## Supporting Information

S1 Text Supplementary material. (PDF)
