# Supplementary material for: Machine Learning Assisted Design of Highly Active Peptides for Drug Discovery
Source: PLoS Comput Biol. 2015 Apr 7;11(4):e1004074. doi: 10.1371/journal.pcbi.1004074 (PMC4388847; doi:10.1371/journal.pcbi.1004074)
Supplement: S1 Text — (PDF) [file pcbi.1004074.s001.pdf]

Supplementary Material For:

Machine Learning Assisted Design of Highly Active Peptides for Drug Discovery

Sébastien Giguère[1]*, François Laviolette[1], Mario Marchand[1], Denise Tremblay[2], Sylvain Moineau[2], Éric Biron[3], Xinxia Liang[3] and Jacques Corbeil[4]

1 Department of Computer Science and Software Engineering, Université Laval, Québec, Canada
2 Department of Biochemistry, Microbiology and Bioinformatics, Université Laval, Québec, Canada
3 Faculty of Pharmacy, Université Laval, Québec, Canada
4 Department of Molecular Medicine, Université Laval, Québec, Canada
∗ E-mail: Corresponding sebastien.giguere.8@ulaval.ca

Expected outcome of a library given a protocol

The proposed approach makes use of the graph $G^{h_{\mathbf{y}}}$, the protocol P, and a dynamic programming algorithm that exploits recurrences in the factorization of first and second order polynomials. This allows for the efficient computation of τ and β, respectively the first and second moment of $h_{\mathbf{y}}$ when peptides are drawn according to the distribution P:

$$\tau \stackrel{\text{def}}{=} \sum_{\mathbf{x}\in\mathcal{A}^l} P(\mathbf{x}) \cdot h_{\mathbf{y}}(\mathbf{x})$$
$$\beta \stackrel{\text{def}}{=} \sum_{\mathbf{x}\in\mathcal{A}^l} P(\mathbf{x}) \cdot h_{\mathbf{y}}(\mathbf{x})^2 \,.$$

Thus, the average and variance predicted bioactivity of peptides synthesized by the protocol are then respectively given by τ and $\beta - \tau^2$.

To compute these quantities efficiently, the dynamic programming algorithm, given in Algorithm 1, uses the following recurrence relations:

$$\sum_{i=1}^{n} x_i = x_n + \sum_{i=1}^{n-1} x_i \,, \tag{1}$$

and

$$\left(\sum_{i=1}^{n} x_i\right)^2 = \left(\sum_{i=1}^{n-1} x_i\right)^2 + 2x_n\left(\sum_{i=1}^{n-1} x_i\right) + x_n^2 \,. \tag{2}$$

Moreover, each node of the graph $G^{h_{\mathbf{y}}}$ has the following additional variables.

- $\boldsymbol{\tau}[s,i]$ for the expected length of paths from the source node λ to the node (s,i).
- $\boldsymbol{\beta}[s,i]$ for the expected squared length of paths from the source node λ to the node (s,i).
- $\boldsymbol{\rho}[s,i]$ is the probability of having the k-mers s at position i.

After the execution of Algorithm 1, the values of τ and β are respectively given by $\boldsymbol{\tau}[t]$ and $\boldsymbol{\beta}[t]$ for the sink node t.

Algorithm 1 Algorithm for computing split and pool synthesis statistics using $G^{h_{\mathbf{y}}}$ and $\mathcal{P}$

$\boldsymbol{\tau}, \boldsymbol{\beta}, \boldsymbol{\rho}$: arrays with $n|\mathcal{A}|^k + 2$ entries initialized to 0

```
for all s ∈ A^k do                                          ▷ Edges leaving the source node
    τ[s, 1] ← P_1(s_1)W(s, 1)
    β[s, 1] ← P_1(s_1)W(s, 1)^2
    ρ[s, 1] ← P_1(s_1)
end for

for i = 2 → n do
    for all s ∈ A^k do
        for all a ∈ A do                                    ▷ Visiting edge ((s, i − 1), (s′, i))
            s′ ← s_2, . . . , s_k, a
            τ[s′, i]+ = P_i(s_2)(τ[s, i − 1] + ρ[s, i − 1]W(s′, i))
            β[s′, i]+ = P_i(s_2)(β[s, i − 1] + ρ[s, i − 1]W(s′, i)^2 + 2τ[s, i − 1]W(s′, i))
            ρ[s′, i]+ = P_i(s_2)ρ[s, i − 1]
        end for
    end for
end for

for all s ∈ A^k do                                          ▷ Edges heading to the sink node
    r ← ∏_{i=1}^{k−1} P_{n+i}(s_{i+1})
    τ[t]+ = r(τ[s, n] + ρ[s, n]W_t(s))
    β[t]+ = r(β[s, n] + ρ[s, n]W_t((s, n), t)^2 + 2τ[s, n]W_t(s))
    ρ[t]+ = rρ[s, n]
end for

return τ[t], β[t] − τ[t]^2                                  ▷ Return average and variance
```

Peptide synthesis, bacterial strains and minimal inhibitory concentration assay

Peptides were synthesized on a Prelude Peptide Synthesizer (Protein Technologies Inc, AZ) using standard Fmoc solid phase peptide chemistry [1]. The synthesis was performed on Rink Amide AM resin and the amino acid couplings achieved with HCTU (2-(6-Chloro-1H-benzotriazole-1-yl)-1,1,3,3-tetramethylaminium hexafluorophosphate) and NMM (N-methylmorpholine). The peptides were cleaved from the resin using a mixture of 95% trifluoroacetic acid, 2.5% triisopropylsilane, 2.5% water for 3h at room temperature and precipitated in cold diethyl ether. After triturating for 2 min, the peptides were collected upon centrifugation and decantation of the ether. The peptides were purified on a Vydac C18 reversed-phase HPLC column (22×250 mm, 5μm) over 20 min using a linear gradient of $10-90\%$ acetonitrile with 0.1% trifluoroacetic acid at a flow rate of 10 mL/min with optical density monitoring at 220 nm. The collected fractions were lyophilised and the identity and purity of the peptides assessed by analytical HPLC and MALDI-TOF mass spectrometry. Peptides were obtained in good yields and with purity greater than 90%.

Escherichia coli K12 MG1655 and *Staphylococcus aureus* 68 (HER1049) were obtained from the Félix d'Hérelle Reference Center for Bacterial Viruses of Université Laval (http://www.phage.ulaval.ca). Both strains were grown in Trypticase soy broth with agitation at 37°C. The minimal inhibitory concentration assay was performed as described in [2] and broth microdilution protocol performed in 96-well plates. The bacterial strains were grown overnight at 37°C with aeration and diluted to a final concentration of 5×10^5 cfu/ml in the assay. The peptides were diluted in sterile water and were tested at the following concentrations: $0, 1, 2, 4, 8, 16$ and 32 μg/ml. The optical density (600nm) was followed every 30 minutes for 24 hours with a Synergy 2 plate reader (BioTek Instruments, Inc.).

References

1. Wellings DA, Atherton E (1997) [4] standard fmoc protocols. Methods in enzymology 289: 44–67.

2. Wiegand I, Hilpert K, Hancock RE (2008) Agar and broth dilution methods to determine the minimal inhibitory concentration (mic) of antimicrobial substances. Nature protocols 3: 163–175.
